# Supplementary material for: Microbial Life Inside Posidonia Seeds: Beneficial Endophytes and Implications for Marine Plant Health
Source: Microbiologyopen. 2026 Mar 5;15(2):e70259. doi: 10.1002/mbo3.70259 (PMC12961358; doi:10.1002/mbo3.70259)
Supplement: Supplementary file 1 — Appendix 1. [file MBO3-15-e70259-s002.docx]

**Appendix 1.** Weight of *Posidonia oceanica* seeds, total colonies counted and Colony Forming Unit (CFU)/g of fresh material on SGY and NA media

| Seed name | Location | Seed weight (g) | Total microbial colonies counted (plate with dilution zero) | | CFU/g | |
| --- | --- | --- | --- | --- | --- | --- |
|  |  |  | SGY | NA | SGY | NA |
| SN1 | San Nicola | 0.327 | 301 | 202 | 295.84 | 198.54 |
| SN2 | San Nicola | 0.205 | 104 | 6 | 66.52 | 3.84 |
| SN3 | San Nicola | 0.174 | 0 | 0 | 0.00 | 0.00 |
| SN4 | San Nicola | 0.312 | 0 | 0 | 0.00 | 0.00 |
| SN5 | San Nicola | 0.293 | 0 | 0 | 0.00 | 0.00 |
| SN6 | San Nicola | 0.054 | 2 | 14 | 0.35 | 2.48 |
| SN7 | San Nicola | 0.211 | 102 | 87 | 67.03 | 57.17 |
| SN8 | San Nicola | 0.363 | 2 | 2 | 2.16 | 2.16 |
| E1 | Erice | 0.220 | 1 | 0 | 0.68 | 0.00 |
| E2 | Erice | 0.266 | 2 | 0 | 1.63 | 0.00 |
| E3 | Erice | 0.182 | 0 | 0 | 0.00 | 0.00 |
| E4 | Erice | 0.286 | 35 | 30 | 30.46 | 26.11 |
| E5 | Erice | 0.089 | 7 | 5 | 2.02 | 1.44 |
| C1 | Cornino | 0.320 | 376 | 352 | 362.41 | 339.28 |
| C2 | Cornino | 0.292 | 47 | 3 | 41.69 | 2.66 |
| C3 | Cornino | 0.145 | 248 | 260 | 114.34 | 119.87 |
| C4 | Cornino | 0.218 | 6 | 0 | 4.06 | 0.00 |
| C5 | Cornino | 0.186 | 7 | 0 | 4.09 | 0.00 |
| C6 | Cornino | 0.163 | 300 | 300 | 154.60 | 154.60 |
| C7 | Cornino | 0.217 | 0 | 0 | 0.00 | 0.00 |
| C8 | Cornino | 0.279 | 0 | 15 | 0.00 | 12.76 |
| C9 | Cornino | 0.258 | 0 | 1 | 0.00 | 0.79 |
| C10 | Cornino | 0.239 | 2 | 3 | 1.48 | 2.21 |
| SC1 | Sciacca | 0.187 | 2 | 2 | 1.17 | 1.17 |
| SC2 | Sciacca | 0.225 | 2 | 0 | 1.40 | 0.00 |
| SC3 | Sciacca | 0.189 | 1 | 0 | 0.59 | 0.00 |
| SC4 | Sciacca | 0.210 | 2 | 1 | 1.31 | 0.65 |
| SC5 | Sciacca | 0.192 | 5 | 3 | 3.01 | 1.80 |
| SC6 | Sciacca | 0.410 | 0 | 2 | 0.00 | 2.40 |
| SC7 | Sciacca | 0.240 | 2 | 0 | 1.48 | 0.00 |
| SC8 | Sciacca | 0.389 | 1 | 2 | 1.15 | 2.30 |
| SC9 | Sciacca | 0.148 | 2 | 3 | 0.94 | 1.41 |
| SC10 | Sciacca | 0.316 | 2 | 0 | 1.91 | 0.00 |
| SC11 | Sciacca | 0.212 | 115 | 0 | 75.90 | 0.00 |
| SC12 | Sciacca | 0.195 | 33 | 0 | 20.14 | 0.00 |
| M1 | Marsala | 0.297 | 0 | 0 | 0.00 | 0.00 |
| M2 | Marsala | 0.218 | 0 | 0 | 0.00 | 0.00 |
| M3 | Marsala | 0.169 | 0 | 0 | 0.00 | 0.00 |
| M4 | Marsala | 0.333 | 1 | 1 | 1.00 | 1.00 |
| M5 | Marsala | 0.314 | 1 | 0 | 0.95 | 0.00 |
| M6 | Marsala | 0.170 | 0 | 0 | 0.00 | 0.00 |
| M7 | Marsala | 0.288 | 234 | 203 | 204.96 | 177.81 |
| M9 | Marsala | 0.297 | 11 | 8 | 9.91 | 7.21 |
| M10 | Marsala | 0.311 | 28 | 38 | 26.30 | 35.69 |
