# Supplementary material for: Microbial Life Inside Posidonia Seeds: Beneficial Endophytes and Implications for Marine Plant Health
Source: Microbiologyopen. 2026 Mar 5;15(2):e70259. doi: 10.1002/mbo3.70259 (PMC12961358; doi:10.1002/mbo3.70259)
Supplement: Supplementary file 2 — Appendix 2. [file MBO3-15-e70259-s001.docx]

**Appendix 2**: Morphological characterization of the 44 selected endophytes isolated from *Posidonia oceanica* seeds collected in the five localities. Cornino, Erice, Marsala, Sciacca, San Nicola.

| **Isolate** | **Location** | **Colony shape** | **Colony elevation** | **Colony surface** | **Colony margin** | **Colony colour** | **Halo** | **Medium** | **Morphotypes** |
| --- | --- | --- | --- | --- | --- | --- | --- | --- | --- |
| Bsp-PoC1 | Cornino | circular | convex | smooth | even | creamy | no | SGY | I |
| Vt-PoC1 | Cornino | circular | flat | smooth | even | white, shiny | no | SGY | II |
| Vsp-PoC2 | Cornino | rhizoid | flat | smooth | curled | creamy center and  white around | no | SGY | III |
| Vt-PoC2 | Cornino | circular | flat | smooth | even | white, shiny | no | SGY | II |
| Vt-PoC4 | Cornino | circular | flat | smooth | even | white, shiny | no | SGY | II |
| Vz-PoC5 | Cornino | rhizoid | flat | smooth | curled | creamy | no | SGY | IV |
| Vz-PoC6 | Cornino | circular | flat | smooth | even | creamy | no | SGY | V |
| Vsp-PoC8 | Cornino | circular | thin | smooth | even | beige, opaque | no | NA | VI |
| Kp-PoC9 | Cornino | circular | umbonate | smooth | even | creamy center and white around, opaque | no | NA | VII |
| Mbi-PoC10 | Cornino | circular | convex | smooth | even | creamy, shiny | yes | SGY | VIII |
| Vsp-PoE2 | Erice | circular | flat | concentric | wavy | creamy | no | SGY | IX |
| Vsp-PoE4 | Erice | circular | flat | concentric | wavy | creamy center and white around | no | SGY | X |
| Vn-PoE4 | Erice | circular | flat | smooth | even | white, shiny | yes | SGY | XI |
| Se-PoE5 | Erice | circular | flat | smooth | even | yellow - orange | no | SGY | XII |
| Sw-PoE5 | Erice | circular | flat | smooth | even | creamy | no | SGY | V |
| Lysp-PoM4 | Marsala | circular | flat | smooth | wavy | white, shiny | yes | SGY | XIII |
| Vsp-PoM7 | Marsala | circular | flat | smooth | even | white, shiny | no | SGY, NA | II |
| Vg-PoM9 | Marsala | circular | umbonate | smooth | even | white, opaque | no | NA | XIV |
| Vz-PoM10 | Marsala | circular | flat | smooth | even | white, shiny | no | SGY | II |
| Vsp-PoM10 | Marsala | circular | flat | smooth | even | creamy | yes | SGY | XV |
| Vsp-PoSC1 | Sciacca | circular | flat | smooth | even | creamy | yes | NA | XV |
| Vz-PoSC1 | Sciacca | rhizoid | flat | smooth | curled | creamy | no | SGY | IV |
| Vz-PoSC2 | Sciacca | circular | flat | smooth | even | white, shiny | no | SGY | II |
| Mr-PoSC2 | Sciacca | circular | flat | concentric | even | creamy, shiny | no | SGY | XVI |
| Vsp-PoSC3 | Sciacca | circular | flat | smooth | even | white, shiny | no | SGY | II |
| Mr-PoSC4 | Sciacca | circular | flat | concentric | even | creamy, shiny | no | SGY, NA | XVI |
| Sp-PoSC5 | Sciacca | circular | flat | smooth | even | dark creamy | no | SGY | XVII |
| Se-PoSC5 | Sciacca | circular | flat | smooth | even | yellow - orange | no | SGY | XII |
| Sw-PoSC5 | Sciacca | circular | flat | smooth | even | creamy and white | no | SGY | XVIII |
| Vt-PoSC6 | Sciacca | circular | flat | smooth | even | creamy – white, transparent | no | NA | XIX |
| Se-PoSC7 | Sciacca | circular | flat | smooth | even | yellow - orange | no | SGY | XII |
| Mr-PoSC8 | Sciacca | circular | flat | concentric | even | creamy, shiny | no | SGY, NA | XVI |
| Aa-PoSC9 | Sciacca | circular | flat | smooth | even | yellow | no | NA | XX |
| Sw-PoSC9 | Sciacca | circular | flat | smooth | even | creamy | no | SGY | V |
| Mr-PoSC10 | Sciacca | circular | flat | concentric | even | creamy, shiny | no | SGY | XVI |
| Vk-PoSC11 | Sciacca | rhizoid | convex | smooth | curled | white | yes | SGY | XXI |
| Vsp-PoSC11 | Sciacca | circular | flat | smooth | even | white, shiny | no | SGY | II |
| Sw-PoSN6 | San Nicola | circular | flat | smooth | even | creamy | no | NA | V |
| Se-PoSN6 | San Nicola | circular | flat | smooth | even | yellow | no | SGY | XX |
| Hmsp-PoSN7 | San Nicola | circular | flat | smooth | even | orange | no | SGY | XXII |
| Cn-PoSN8 | San Nicola | filamentous | flat | smooth | curled | creamy, opaque | no | NA | XXIII |
| Vn-PoSN8 | San Nicola | circular | flat | smooth | even | white, shiny | yes | SGY | XI |

| **Isolate** | **Location** | **Colony shape** | **Colony colour** | **Aerial mycelium** | **Exudate** | **Growth rate** | **Medium** | | **Morphotype** |
| --- | --- | --- | --- | --- | --- | --- | --- | --- | --- |
| Pm-PoE1 | Erice | irregular circular | brown | powdery to suede-like | no | fast | | SGY | XXV |
| Hpsp-PoSN1 | San Nicola | irregular circular | white | feathery | no | slow | | SGY | XXIV |
